# Non-Hematopoietic and Hematopoietic SIRPα Signaling Differently Regulates Murine B Cell Maturation in Bone Marrow and Spleen

**DOI:** 10.1371/journal.pone.0134113

**Published:** 2015-07-29

**Authors:** Shrikant Shantilal Kolan, Kristina Lejon, Cecilia Koskinen Holm, Rima Sulniute, Pernilla Lundberg, Takashi Matozaki, Per-Arne Oldenborg

**Affiliations:** 1 Department of Integrative Medical Biology, Umeå University, Umeå, Sweden; 2 Department of Clinical Microbiology, Section for Immunology, Umeå University, Umeå, Sweden; 3 Department of Odontology, Umeå University, Umeå, Sweden; 4 Department of Biochemistry and Molecular Biology, Division of Molecular and Cellular Signaling, Kobe University Graduate School of Medicine, Kobe, Japan; Charles P. Darby Children's Research Institute, UNITED STATES

## Abstract

B lymphocyte development occurs in the bone marrow, while final differentiation and maturation can occur in both the bone marrow and the spleen. Here we provide evidence that signal regulatory protein α (SIRPα), an Ig-superfamily ITIM-receptor expressed by myeloid but not by lymphoid cells, is involved in regulating B cell maturation. Lack of SIRPα signaling in adult SIRPα-mutant mice resulted in a reduced maturation of B cells in the bone marrow, evident by reduced numbers of semi-mature IgD^+^IgM^hi^ follicular type-II (F-II) and mature IgD^+^IgM^lo^ follicular type-I (F-I) B cells, as well as reduced blood B cell numbers. In addition, lack of SIRPα signaling also impaired follicular B cell maturation in the spleen. Maturing BM or splenic B cells of SIRPα-mutant mice were found to express higher levels of the pro-apoptotic protein BIM and apoptosis was increased among these B cells. Bone marrow reconstitution experiments revealed that the B cell maturation defect in bone marrow and blood was due to lack of SIRPα signaling in non-hematopoietic cells, while hematopoietic SIRPα signaling was important for follicular B cell maturation in the spleen. Adding on to our previous findings of a stromal cell defect in SIRPα-mutant mice was the finding that gene expression of receptor activator of nuclear factor-ĸB ligand (RANKL) was significantly lower in cultured bone marrow stromal cells of SIRPα mutant mice. These data suggest a novel and opposite contribution of SIRPα signaling within non-hematopoietic and hematopoietic cells, respectively, to maintain B cell maturation and to prevent apoptosis in the bone marrow and spleen of adult mice.

## Introduction

B lymphocytes are generated from pluripotent and self-renewing hematopoietic stem cells in the bone marrow (BM) after birth [1]. Newly formed surface IgM^+^ (sIgM^+^) immature B cells emigrate from the BM to the spleen via blood, where different transitional stages primarily leads to differentiation into either mature recirculating follicular B cells (FoB) or marginal zone B cells (MZB) [2,3]. However, immature sIgM^+^ B cells can also directly mature into IgD^hi^ follicular B cells in the BM itself by first becoming semi-mature IgD^+^IgM^hi^ B cells (corresponding to splenic follicular type-II cells) and then fully mature IgD^+^IgM^lo^ B cells (corresponding to splenic follicular type-I cells) [4–6].

B cell commitment and development take place in a complex BM microenvironment which consists of a diverse network of stromal cells. These BM stromal cells create specialized niches and affect proliferation and differentiation of B lineage cells by providing requisite factors essential for B cell development, of which CXC-chemokine ligand 12 (CXCL-12), interleukin-7 (IL-7) and receptor activator of nuclear factor-ĸB ligand (RANKL) have been proposed to play a major role [7,8]. However, later findings have shown that mice with a B cell-specific deletion of the RANKL-receptor RANK have normal B cell development and maturation [9]. Thus, RANKL does not seem to have a direct role in B cell development and maturation.

Follicular dendritic cells (FDCs) and fibroblastic reticular cells (FRCs) are the two main stromal cell subsets located within the B cell follicles of the splenic white pulp, where FDCs are in majority in adult murine spleens. In contrast, FRCs is the most abundant stromal cell type in the T cell rich periarteriolar lymphocyte sheaths (PALS) [10,11]. Specific cytokines secreted by these two stromal cell subsets are important to segregate and support the homeostasis of B and T cells in the spleen. FRCs produce CCL19 and CCL21, which by binding to the receptor CCR7 on T cells mediate T cell migration into the PALS. Directed migration of B cells into B cell follicles is mediated by CXCL13, produced by FDCs and recognized by the B cell receptor CXCR5 [10,12]. FRCs and FDCs also produce factors that promote the survival of lymphocytes. B cell maturation and survival is strictly dependent on the B cell activating factor BAFF, whereas IL-7 promotes the survival and proliferation of T cells [13] as well as the development and survival of splenic follicular B cells [14]. Although BAFF can be produced by both hematopoietic cells (e.g. monocytes, macrophages, dendritic cells and neutrophils) and non-hematopoietic stromal cells, it has been shown that BAFF produced by radiation-resistant stromal cells is required for B cell homeostasis and survival [15]. This function has been attributed to FDCs, however, recent findings have shown that BAFF produced by FRCs and not FDCs is required to maintain B cell homeostasis in secondary lymphoid organs [16].

Signal regulatory protein alpha (SIRPα) is a cell surface Ig-family ITIM-receptor highly expressed in myeloid cells, but not in lymphoid cells, preferentially serving to negatively regulate functional activation (e.g. phagocytosis or cell migration) [17–19]. The ubiquitously expressed Ig-family cell surface glycoprotein CD47 is the only know cellular ligand of SIRPα [20]. Since CD47 can also signal, preferentially through Gα_i_, the CD47-SIRPα interaction can mediate bi-directional signaling upon cell-cell contact [21]. In addition to CD47, surfactant proteins-A and-D are also ligands of SIRPα and may negatively regulate inflammatory activation of alveolar macrophages [22]. Studies of mutant mice, lacking most of the cytoplasmic signaling domain of SIRPα, have shown that signaling through this receptor is important to maintain normal numbers of splenic dendritic cells and T cells [23,24]. Our previous studies showed that spleens of SIRPα-mutant mice had a marked reduction in FRCs in the T cell area and a reduced expression of CCL19, CCL21 and IL-7 [24]. By investigating *cd47* ^*-/-*^ mice, we recently found that stromal cells also express SIRPα, that its tyrosine phosphorylation is dependent on CD47 and that SIRPα is required for BM stromal cell osteoblastic differentiation and RANKL production [25]. Thus, SIRPα signaling in the BM as well as in splenic stromal cells appears to be of importance for regulating their numbers and function. Given the requirement of SIRPα signaling for normal function of stromal cells and the importance of these cells for B cell development, we here investigated B cell homeostasis in SIRPα signaling-mutant mice.

## Materials and Methods

### Mice

Male and female C57BL/6 Ly5.2 *SIRPα-*mutant mice, lacking most of the SIRPα cytoplasmic domain has been previously described [26]. C57BL/6 Ly5.1 mice were from Taconics, Italy. All the experiments were performed in strict compliance with relevant Swedish and institutional laws and guidelines and were approved by the Umeå Research Animal Ethics Committee (A14-12). All injections in mice were made under isofluoran anesthesia. Animals were euthanized by CO_2_ asphyxiation and cervical dislocation before organs were surgically removed for further analysis. In bone marrow reconstitution experiments, mice were followed daily to assure that the weight loss did not exceed 10% or that the animals suffered from dehydration. Reconstituted animals were kept in IVC-cages to reduce the risk of infection.

### Reagents

PE-conjugated anti-CD23 (eBioscience-B3B4), FITC-conjugated anti-IgM (Sigma-μ chain specific) or brilliant violet (BV) 421-conjugated anti-IgM (BD pharmingen-R6-60.2), APC-conjugated anti-CD19 (BD bioscience-1D3), FITC-conjugated anti-CD3ε (Immunotools-145-2C11), APC-conjugated anti-CD11b (Biolegend- M1/70) and PE-conjugated anti-Gr-1(Immunotools- RB6-8C5) were used for flow cytometric analysis of blood leukocytes. Biotinylated anti-B220 (eBioscience-RA3-6B2) or Alexa 647-conjugated anti-B220 (BD Pharmingen- RA3-6B2), PE-conjugated anti-IgD (Biolegend-clone 11-26c.2a), FITC-conjugated anti-IgM (Sigma-μ chain specific) or BV 421-conjugated anti-IgM (BD Pharmingen- R6-60.2), FITC- conjugated anti-CD21 (BD Pharmingen- eBio8D9), PE-conjugated anti-CD23 (eBioscience-B3B4), APC-conjugated anti-CD19 (BD bioscience-1D3) or Alexa 700-conjugated anti-CD19 (BD Pharmingen-1D3) were used for flow cytometric analysis of BM or spleen cells. Biotinylated antibodies were detected with APC-conjugated streptavidin (Immunotools) or BV 605-conjugated streptavidin (BioLegend). Rat anti-mouse SIRPα (mAb P84; rat IgG1; a generous gift from Dr.Carl Lagenaur, university of Pittsburgh) was purified and conjugated to Alexa 488 as previously described [18].

### Blood cell analysis

Peripheral blood was obtained by vein puncture, collected into heparinized capillary tubes and diluted into PBS + 5 mM EDTA. After RBC lysis, samples were incubated with fluorochrome-conjugated antibodies followed by addition of fluorescent beads (Countbright absolute counting beads, Life Technologies) to calculate absolute cell numbers. Samples were analyzed by FACS Calibur or LSR II (BD biosciences) and by using Cell Quest or FACSDiva software (BD biosciences).

### Bone marrow and spleen analysis

Single cell suspensions were prepared from BM by flushing femur and tibia with PBS using 27-G (BD) needles, or from spleens by mechanical disruption. Cells were filtered using a 70-μm cell strainer (BD) and erythrocytes were lysed with ACK. Erythrocyte-free cell suspensions were incubated with Fc-block (mAb 2.4G2) before being labeled with antibodies as indicated in the figures. For analysis of B cell BIM protein expression, splenic or BM B cells were stained with the indicated mAbs to enable separation of specific B cell subsets, washed, fixed in fresh 2% paraformaldehyde and permeabilized with 0.1% Triton X100. After washing, cells were stained with an Alexa 647-conjugated anti-BIM mAb (Novus biotech 151–149) and washed twice with FACS buffer. All analyzes were done using FACS Calibur or LSR II (BD biosciences) and by using Cell Quest or FACSDiva software (BD biosciences).

### Apoptosis assay

Erythrocyte-free cell suspensions from BM or spleens were labeled with antibodies as indicated in the figures. Before analysis, cells were incubated with FITC-conjugated Annexin V for 15–30 min and the fraction of Annexin V^+^ B cells within each B cell population was quantified by flow cytometry FACS LSR II (BD biosciences) and by using FACSDiva software (BD biosciences).

### Cell cycle analysis

Erythrocyte-free cell suspensions from BM or spleens were labeled with antibodies as indicated in the figures. One μl of Vybrant DyeCycle Ruby Stain (Life Technologies; final concentration 5μM) was added to 0.5 ml of cell suspension with a cell concentration 5×10^5^ cells/ml. Samples were incubated at 37°C for 15–30 minutes in the dark according to the manufacturer’s instructions. Samples were analyzed using FACS LSR II (BD biosciences) and by using FACSDiva software (BD biosciences).

### Bone marrow reconstitution

Ly5.2 wild-type or SIRPα-mutant recipient mice were lethally irradiated by exposed to 5 Gy at two occasions separated by three hours. After a three hour rest, mice were intravenously injected with 5x10^6^ BM cells isolated from Ly5.1 wild-type mice. Conversely, lethally irradiated Ly5.1 wild-type mice were reconstituted with BM cell isolated from Ly5.2 wild-type or SIRPα-mutant mice. Twenty weeks after BM transplantation, peripheral blood, BM and spleen were subjected to flow cytometric analysis. In these experiments, more than 97% of all BM, blood or splenic leukocytes were of donor origin.

### Bone marrow stromal cell culture

Preparation of bone marrow stromal cell cultures has been described previously [25]. In brief, BM cells were isolated by flushing femurs and tibiae of 16 wks old wild-type or SIRPα mutant mice. The cells were cultured in 60cm^2^ culture dishes (Nunc) with α-MEM media with 10% FCS (Invitrogen), L-glutamine and antibiotics (Sigma Aldrich) for 12 days. After 12 days, cells were lysed for RNA isolation.

### Quantitative Real-time PCR

Isolation of total RNA and synthesis of cDNA from mRNA from BM stromal cells was done as previously described [25]. Using the standard curve method, the relative expression of *rankl* in BM stromal cell was calculated based on the threshold cycle (Ct) values of *rankl* gene (Taqman gene expression assay) and the Ct values of the endogenous control β-actin by using Taqman ABI PRISM 7900 HTSequence Detection system.

mRNA was extracted from FACS sorted splenic follicular B cells CD19^+^CD21^int^CD23^hi^; >95% purity) using RNeasy Mini kit (Qiagen) according to the manufacturer’s instructions. Total RNA (200 ng) was reverse transcribed using High capacity cDNA reverse transcription kit (Applied Biosystems). Bcl-2 and BIM (Bcl2l11) mRNA levels were determined by using a ViiA 7 instrument (Applied Biosystems). Ct values were used to calculate relative values and were normalized to that of β-actin. The following Taqman gene expression assays (Applied Biosystems) containing pre-designed primers and probes were used: Bcl-2, (Mm00477631_m1); BIM (Bcl2l11) (Mm00437796_m1); β-actin (NM_007393.1).

### Statistics

Statistical analyses were performed using Students t-test for unpaired analyses. Significance levels are indicated as *P<0.05, **P<0.01 and ***P<0.001.

## Results

### Lack of SIRPα signaling results in a reduced number of circulating B cells

**Fig 1:**
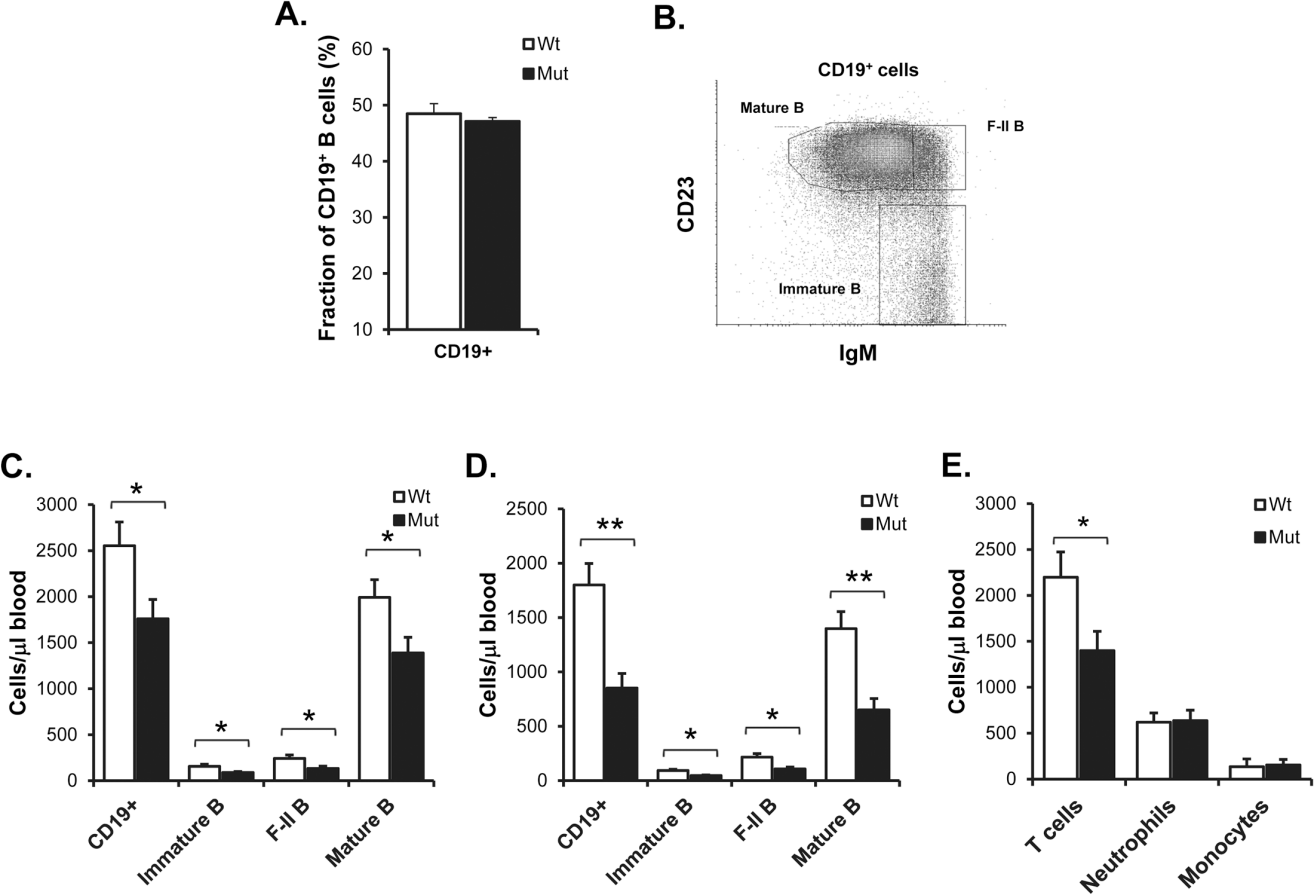
Lack of SIRPα signaling *in vivo* results in reduced numbers of blood B cells. (A) Fraction of CD19^+^ blood B cells in 12 weeks old wild-type (Wt–open bars) or SIRPα-mutant mice (Mut–black bars). (B) CD19^+^ blood B cell subsets were separated based on their expression levels of sIgM and CD23 to identify sIgM^hi^CD23^-/lo^ immature B cells (Immature B), sIgM^hi^CD23^hi^ semi-mature/follicular type-II (F-II B) B cells and sIgM^lo/int^CD23^hi^ mature/F-I (Mature B) B cells. Data shown are the numbers of total CD19^+^, immature, F-II, or mature B cells per microliter of blood in wild-type (Wt–open bars) or SIRPα-mutant mice (Mut–black bars) at (C) 12 weeks or (D) 20 weeks of age. Data are means±SEM for 9 mice/group at 12 weeks and 4–7 mice/group at 20 weeks of age. (E) Absolute numbers of CD4/8^+^ T cells, CD11b^hi^ Gr1^hi^ neutrophils and CD11b^hi^ Gr1^low^ monocytes per microliter of blood in 16 weeks old wild-type (Wt–open bars) or SIRPα-mutant mice (Mut–black bars). Data are means±SEM for 5 mice/group. *P<0.05 and **P<0.01, as compared with that in wild-type mice, using Student’s t-test for unpaired analyses.

We have previously found that the proportion of blood B cells out of total lymphocytes was similar in 6–12 weeks old wild-type and SIRPα-mutant mice [24]. We confirmed this in blood of 12 weeks old wild-type and SIRPα-mutant mice (48.5±1.8% vs. 47.1±0.7%, respectively; P>0.05) (Fig 1A). However, the absolute number of CD19^+^ B cells was reduced by 30%, as compared with that in age-matched wild-type mice (P<0.05, Fig 1C). The discrepancy between a normal B cell frequency and reduced absolute B cell numbers can be explained by the fact that the absolute number of blood T cells was reduced by about 33% in SIRPα-mutant mice (Fig 1E). Since the CD19^+^ blood B cell population contains several separate B cell subsets at different stages of their maturation, we also labeled blood cells with mAbs against sIgM and CD23 to enable identification of sIgM^hi^CD23^-/lo^ immature B cells, sIgM^hi^CD23^hi^ semi-mature/follicular type-II B cells and sIgM^lo/int^CD23^hi^ mature/follicular type-I B cells (Fig 1B). In 12 weeks old SIRPα-mutants, all three B cell subsets were significantly reduced (43%, 45% and 30%, respectively), as compared with that in wild-type mice (Fig 1C). This blood B cell deficit of SIRPα-mutant mice was further enhanced at 20 weeks of age (Fig 1D). To investigate if SIRPα-mutant mice also had a reduced number of myeloid lineage cells in the blood, we quantified the numbers of neutrophils and monocytes in blood of 16 weeks old wild-type or SIRPα-mutant mice. This analysis revealed no differences among neutrophils or monocytes in the two genotypes of mice (Fig 1E). Thus, lack of SIRPα signaling appeared to be of importance to maintain normal numbers of circulating B cells in adult mice.

### Impaired B cell maturation in bone marrow and spleen of SIRPα-mutant mice

**Fig 2:**
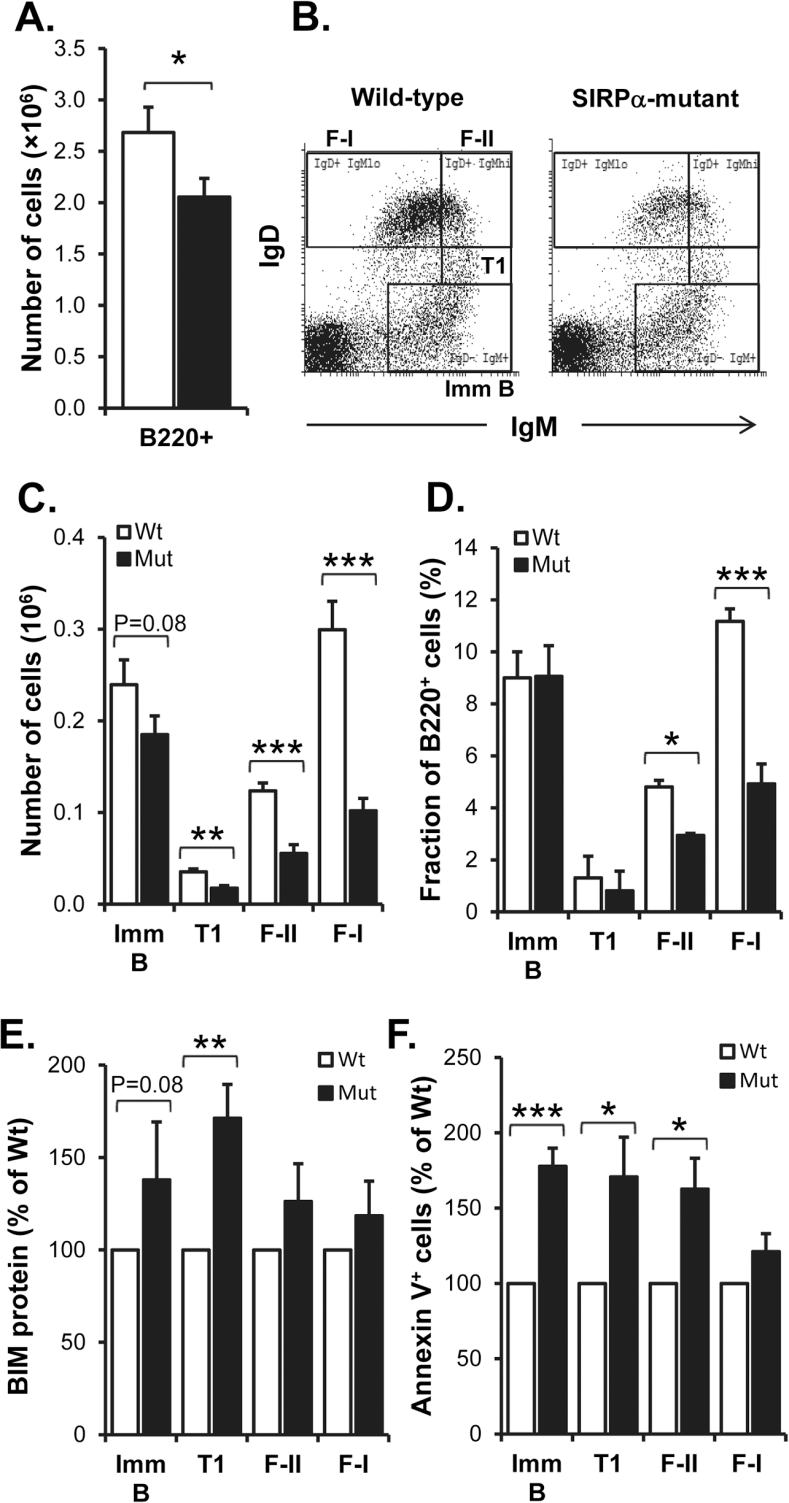
Impaired B cell maturation in the bone marrow of SIRPα-mutant mice. (A) Absolute number of B220^+^ B cells in BM of 12 weeks old wild-type (open bar) or SIRPα-mutant mice (black bar). Data are means±SEM for 9 mice/group. (B) Representative flow cytometry analyses of BM B cell maturation based on expression of IgD and IgM in B220^+^ BM cells of wild-type or SIRPα-mutant mice. (C) Absolute numbers, or (D) relative numbers, of IgD^-^IgM^+^ immature (Imm B), IgD^lo^IgM^hi^ transitional 1 (T1), IgD^+^IgM^hi^ follicular type-II (F-II) and IgD^+^IgM^lo^ follicular type-I (F-I) B cells in BM of 12 weeks old wild-type (open bars) or SIRPα-mutant mice (black bars). Data are means±SEM for 9 mice/group. (E) The relative expression of BIM protein, and (F) the relative increase in annexin V^+^ apoptotic cells, were quantified among BM B cell subsets defined as described in panels C-D. Data are means±SEM for 5 mice/group. *P<0.05, **P<0.01 and ***P<0.001, using Student’s t-test for unpaired analyses.

**Fig 3:**
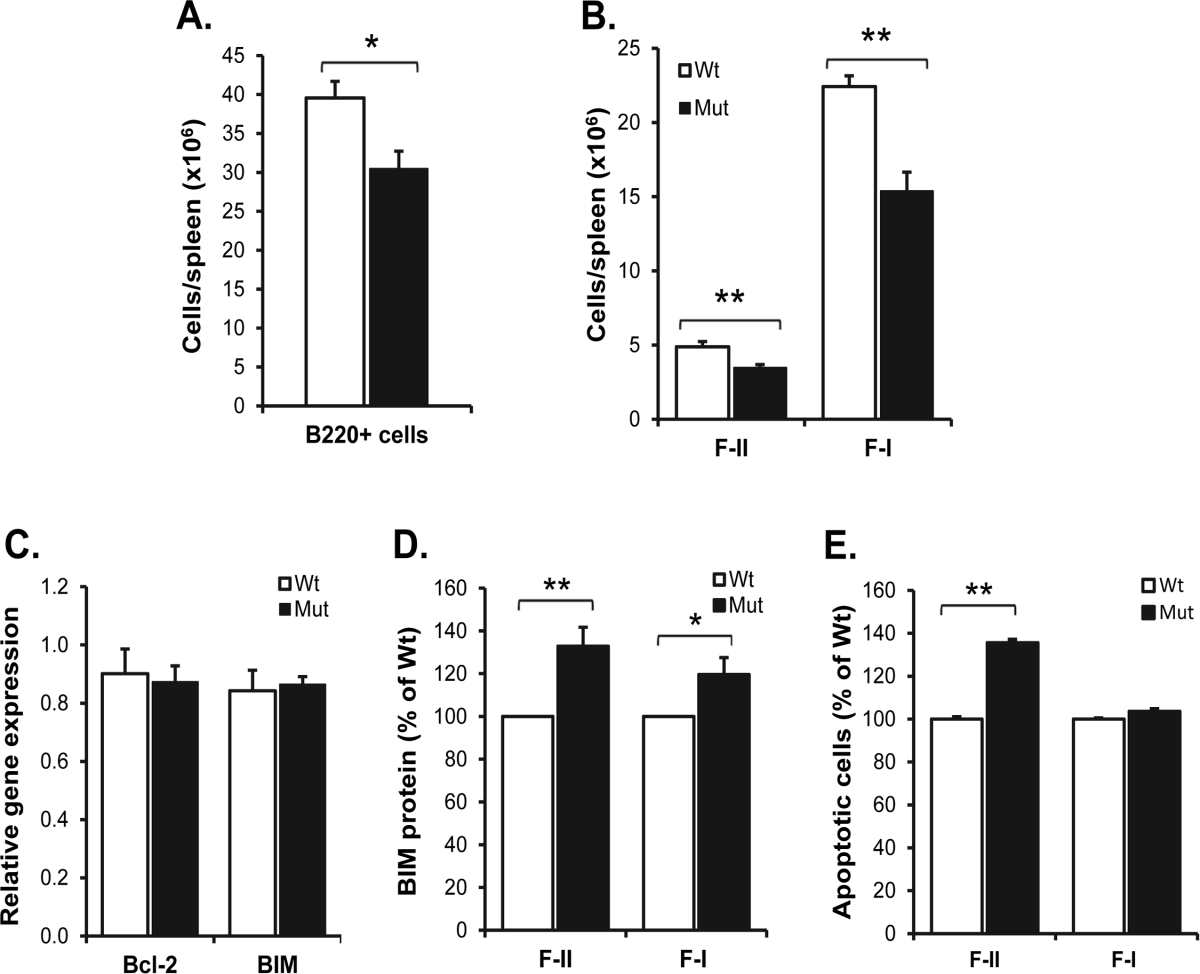
Reduced number of mature B cells in the spleen of SIRPα-mutant mice. (A) Absolute numbers of total B220^+^ B cells in the spleen of 12 weeks old wild-type (open bars) or SIRPα-mutant mice (black bars). (B) Absolute numbers of B220^+^CD21^int^IgD^+^IgM^hi^ follicular type-II (F-II) and B220^+^CD21^int^IgD^+^IgM^lo^ follicular type-I (F-I) B cells in the spleens of 12 weeks old wild-type (open bars) or SIRPα-mutant mice (black bars). Data are means±SEM for 5 mice/group. (C) Relative mRNA levels of Bcl-2 or BIM in sorted splenic CD19^+^CD21^int^CD23^hi^ follicular B cells from 12 weeks old wild-type (open bars) or SIRPα-mutant mice (black bars) were determined by RT-qPCR. Values were normalized to β-actin and depicted as mean±SD of 4 mice per genotype. (D) The relative expression of BIM protein, and (E) the relative increase in annexin V^+^ apoptotic cells, were quantified among BM B cell subsets defined as described in panel B. Data are means±SEM for 5 mice/group. *P<0.05 and **P<0.01, using Student’s t-test for unpaired analyses.

It has been suggested that approximately two thirds of the sIgM^+^ immature B cells generated in the BM will be released into the blood and migrate to the spleen for further maturation, while the remaining immature B cells mature within the BM itself [5,27]. Therefore, we next investigated if the maturation of B cells in the BM was affected in SIRPα-mutant mice. While the total number of BM cells in 13 weeks old SIRPα-mutant mice was similar to that in wild-type mice (S1A Fig), mutants had significantly less B220^+^ B cells (P<0.05, Fig 2A). We found no difference in the absolute numbers of early B lineage Hardy fractions A (B220^+^ IgM^-^ CD43^hi^ CD19^-^), B-C (B220^+^ IgM^-^ CD43^hi^ CD19^+^), or D (B220^+^ IgM^-^ CD43^lo^ CD19^+^) cells in the BM when comparing wild-type and SIRPα-mutant mice (S1B Fig). Further analysis of BM B cell subsets ranging from immature to mature B cells can be made by following the expression levels of IgD and IgM. IgD^-^IgM^+^ immature B cells that mature within the BM first become IgD^lo^IgM^hi^ transitional 1 (T1) B cells, followed by becoming semi-mature IgD^+^IgM^hi^ B cells (corresponding to splenic follicular type-II cells; F-II) and then fully mature IgD^+^IgM^lo^ B cells (corresponding to splenic follicular type-I cells; F-I), (Fig 2B) [4–6]. Using this approach, we found that the reduced amount of IgD^-^IgM^+^ immature B cells found in SIRPα-mutant mice did not reach a higher level of statistical significance (P = 0.075, Fig 2C). However, the numbers of the T1, F-II and F-I subsets were all significantly reduced in the BM of SIRPα-mutant mice (P<0.01, P<0.001 and P<0.001, respectively) (Fig 2C). We also investigated the relative frequency as fractions of the total number of B220^+^ BM cells. This analysis revealed that the fractions of IgD^-^ IgM^+^ immature B cells and IgD^lo^IgM^hi^ T1 B cells were not significantly different from that in wild-type mice (Fig 2D), whereas the fraction of F-II cells was reduced by 40% and that of F-I cells was reduced by 55% (P<0.05 and P<0.001, respectively) (Fig 2D). Since the majority of immature B cells mature in the spleen [5,27], we further investigated the numbers of B220^+^CD21^int^IgD^hi^IgM^hi^ F-II and B220^+^CD21^int^IgD^hi^IgM^lo^ F-I B cells in the spleens of SIRPα-mutant mice. This analysis first revealed that 13 weeks old mutant mice had a significantly reduced number of B220^+^ B cells (P<0.05, Fig 3A). Second, both the F-II and F-I B cell subsets were significantly reduced in SIRPα-mutant spleens, as compared with that in wild-type spleens (P<0.01, Fig 3B). Supporting our previous finding that B cells lack SIRPα expression in secondary lymphoid organs [24], we found that SIRPα was neither expressed by B220^+^ IgM^+^ CD43^-^ B cells in the BM (S2A Fig) nor by splenic FoB cells (S2B Fig). Thus, SIRPα signaling outside the B cell compartment appeared to be required to maintain normal levels of immature B cells in the BM and to support B cell maturation in both the BM and the spleen. B cell survival is regulated by pro-survival proteins (e.g. Bcl-2 and Bcl-X_L_) and pro-apoptotic proteins (e.g. BIM) [28]. To test the hypothesis that there was a reduced survival of mature follicular B cells in mutant spleens, we quantified the mRNA expression levels of Bcl-2 and BIM in FACS-sorted CD19^+^CD21^int^CD23^hi^ mature splenic FoB cells. This analysis showed that there were no differences in gene expression of these two proteins when comparing wild-type and mutant FoB cells (Fig 3C). However, it has been recognized that in lymphocytes, the pro-apoptotic activity of BIM can also be regulated at the posttranslational level [29]. Therefore, we next investigated BIM protein levels in BM or splenic B cells of wild-type or SIRPα-mutant mice. In the BM, we found an increased level of BIM protein in immature and T1 B cells (P = 0.08 and P<0.01, respectively; Fig 2E). In addition, both splenic F-II and F-I B cells showed an increased level of BIM protein (P<0.01 and P<0.05, respectively; Fig 3D). To further investigate the possibility of an increased rate of B cell apoptosis in SIRPα-mutant mice, we quantified the amount of annexin V-positive cells. We found a significantly increased amount of apoptotic immature, T1 and F-II B cells in the BM of mutant mice, as compared with that in wild-type mice (P<0.001, P<0.05 and P<0.05, respectively; Fig 2F). In the spleen, the fraction of apoptotic F-II B cells was significantly increased in SIRPα-mutant mice, as compared with that in wild-type mice (P<0.01; Fig 3E). In contrast, we did not find any differences in cell cycle activity among these B cell subsets in the spleen or BM when comparing wild-type and SIRPα-mutant mice (S3 Fig). Thus, a reduced number of maturing B cells in the BM and spleen of SIRPα-mutant mice appeared to be associated with an increased rate of B cell apoptosis.

### B cell maturation in the bone marrow and spleen shows different requirements for SIRPα signaling within the non-hematopoietic and hematopoietic compartments

**Fig 4:**
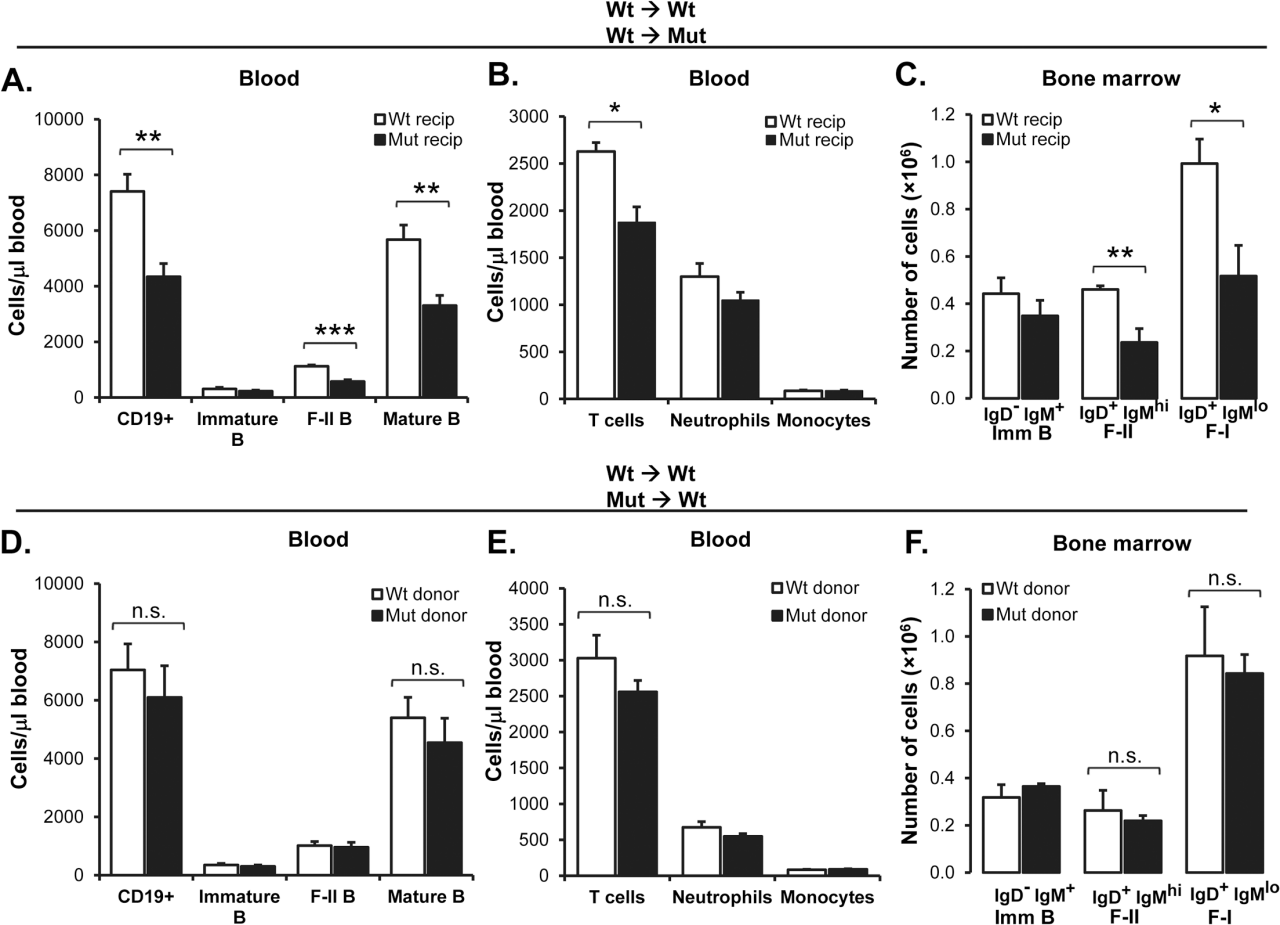
Requirement of non-hematopoietic SIRPα to maintain normal numbers of blood and BM B cells. (A-C) Blood and BM B cell numbers in Ly5.2 wild-type (Wt recip) or SIRPα-mutant (Mut recip) chimeric mice at 20 weeks after lethal irradiation and reconstitution with Ly5.1 wild-type BM cells. (A) Numbers of total CD19^+^, immature, F-II, or mature B cells in blood of wild-type or SIRPα-mutant recipient mice were quantified based on their expression levels of IgM and CD23, as described in the legend to Fig 1. (B) Absolute numbers of CD4/8^+^ T cells, CD11b^hi^ Gr1^hi^ neutrophils and CD11b^hi^ Gr1^low^ monocytes per microliter of blood in wild-type or SIRPα-mutant recipient mice. (C) Immature (IgD^-^IgM^+^), F-II (IgD^+^IgM^hi^) and F-I (IgD^+^IgM^lo^) BM B cells were quantified as described in the legend to Fig 2D. Data are means±SEM for 5 mice per genotype. (D-F) Blood and BM cell numbers in Ly5.1 wild-type mice reconstituted with Ly5.2 wild-type (Wt donor) or SIRPα-mutant (Mut donor) BM. (D) Blood B cell numbers, (E) blood T cell, neutrophil or monocyte numbers, and (F) BM B cell numbers in wild-type recipients of wild-type or SIRPα-mutant BM were quantified as described above. Data are means±SEM for 6 mice per genotype. *P<0.05, **P<0.01 and ***P<0.001, using Student’s t-test for unpaired analyses (n.s. = not significant).

**Fig 5:**
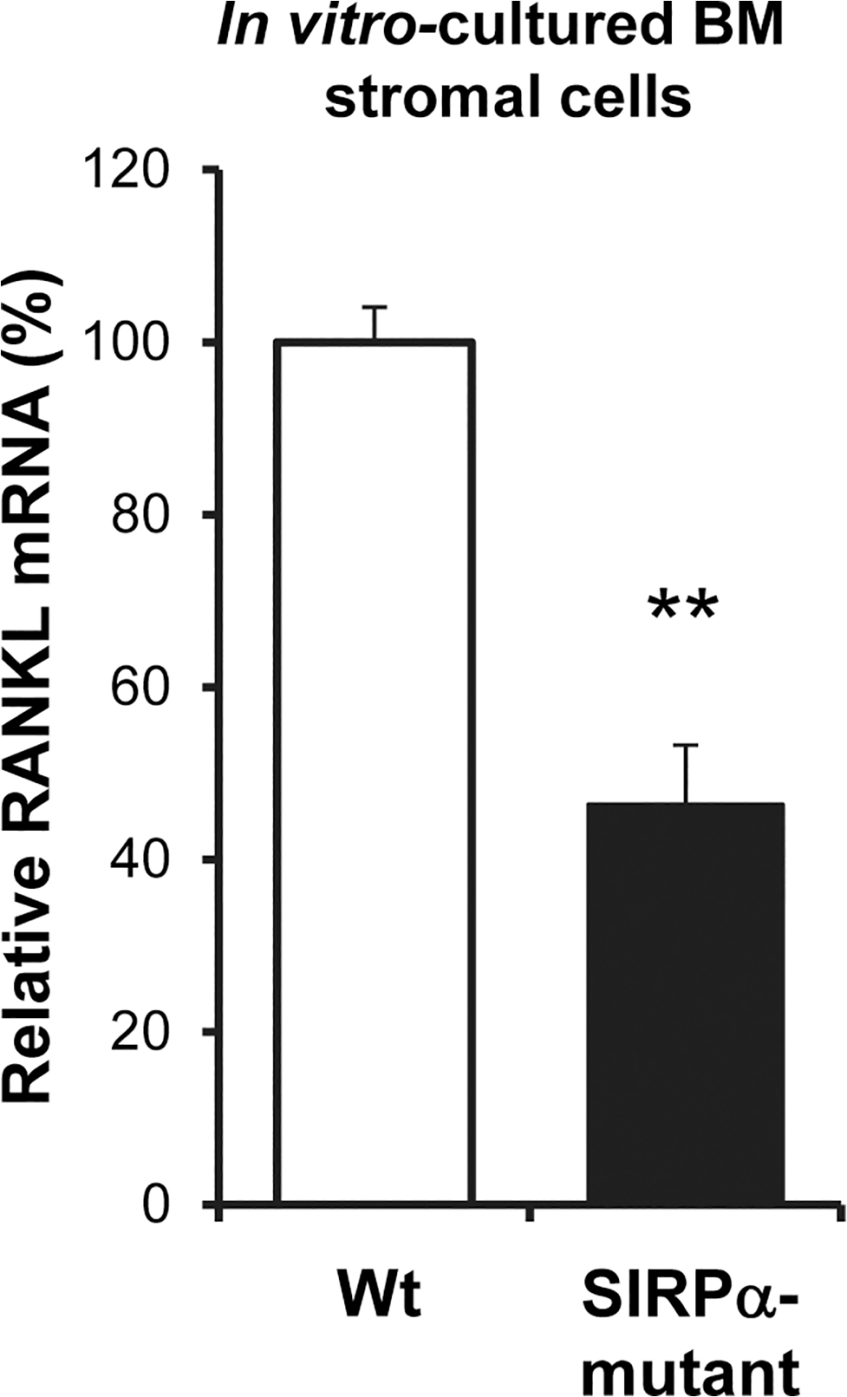
Reduced RANKL gene expression, *in vitro*, in BM stromal cell culture of SIRPα-mutant mice. The mRNA expression of *rankl* in wild-type (Wt–open bar) or SIRPα-mutant mice (Mut–black bar) BM stromal cells cultured for 12 days, was determined by RT-qPCR. Wild-type control was set to 100% and data of mRNA analyses are quantitative values normalized to the house keeping gene β-actin. The *rankl* mRNA expression was analyzed in three separate experiments and the data shown are the means±SEM of 4 samples/group in one representative experiment. **P<0.01, using Student’s t-test for unpaired analyses.

**Fig 6:**
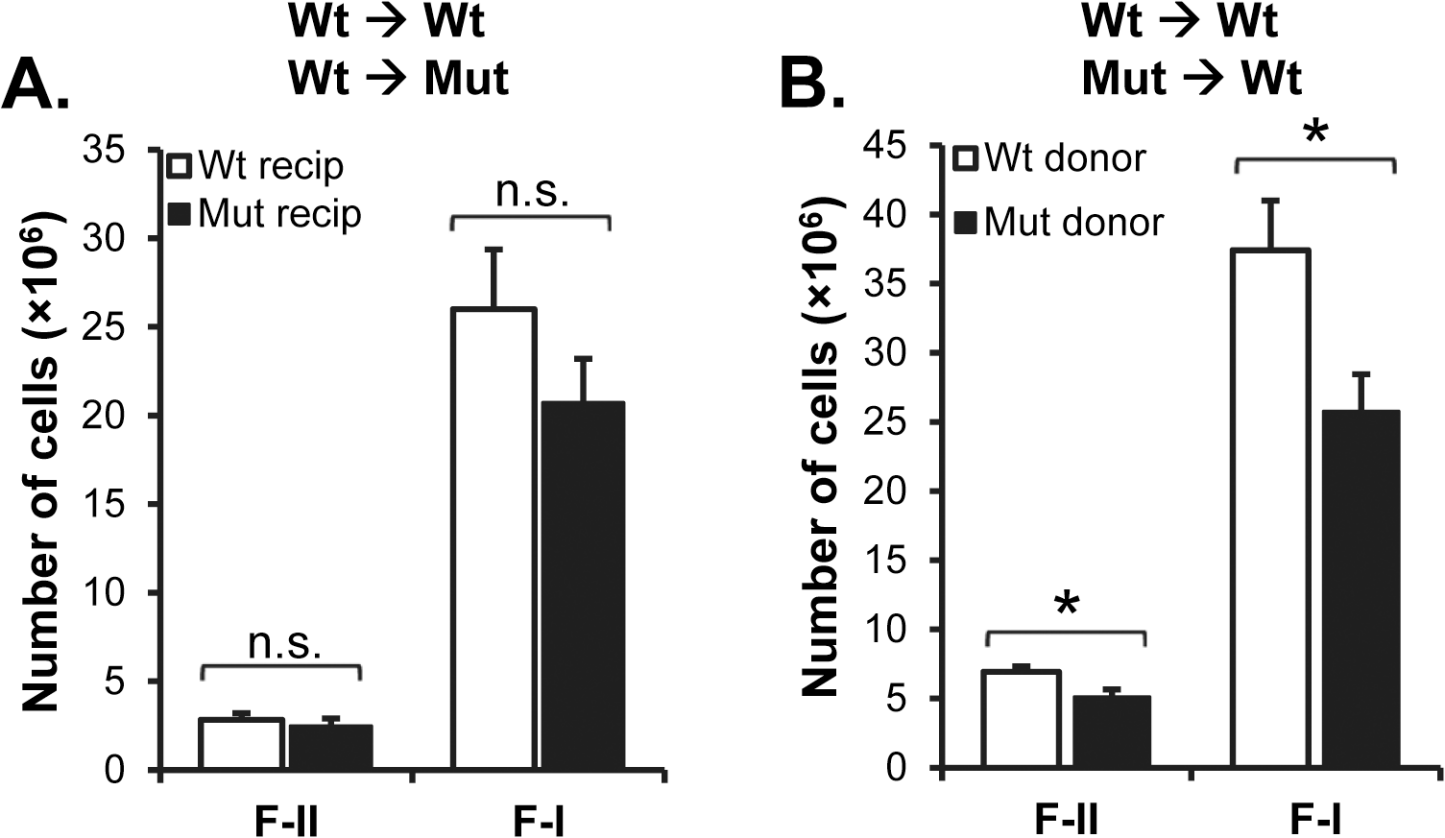
Requirement of hematopoietic SIRPα to maintain normal numbers of splenic mature B cells. Absolute numbers of B220^+^CD21^int^IgD^+^IgM^hi^ follicular type-II (F-II) and B220^+^CD21^int^IgD^+^IgM^lo^ follicular type-I (F-I) B cells in the spleens of (A) Ly5.2 wild-type (Wt recip) or SIRPα-mutant (Mut recip) recipients of Ly5.1wild-type BM (5 mice/group), or (B) Ly5.1wild-type recipients of Ly5.2 wild-type or SIRPα-mutant BM (6 mice/group), at 20 weeks after BM transfer. *P<0.05, using Student’s t-test for unpaired analyses (n.s. = not significant).

In contrast to that in lymphoid cells, SIRPα is expressed by both non-hematopoietic stromal cells [25] and hematopoietic cells [21], such as dendritic cells, which may all be involved in supporting maintenance and maturation of B cells in the BM and spleen [7,30]. To investigate how SIRPα signaling within the hematopoietic and non-hematopoietic compartments contributed to B cell maturation in the BM and spleen, we reconstituted either Ly5.2 wild-type or SIRPα-mutant mice with Ly5.1 wild-type BM cells, or Ly5.1 wild-type mice with Ly5.2 wild-type or mutant BM cells. We then followed the blood B cell numbers in these chimeric mice over time to find a blood B cell phenotype that was similar to the of naïve SIRPα-mutant mice. At 20 weeks after BM transfer, the number of CD19^+^ blood B cells was significantly reduced in SIRPα-mutants receiving wild-type BM, as compared with that in wild-type recipients of wild-type BM (Fig 4A). Using the gating strategy described in Fig 1A, we found that the number of immature B cells was normal whereas that of F-II and mature B cells were significantly reduced in SIRPα-mutant recipients of wild-type BM (P<0.001 and P<0.01, respectively) (Fig 4A). In contrast, all B cell subsets were normal in the blood of wild-type mice receiving either wild-type or mutant BM (Fig 4D). In the blood of SIRPα-mutant recipients of wild-type BM, the number of T cells was significantly reduced (P<0.05), while the numbers of neutrophils or monocytes were similar, as compared with that in wild-type recipients of wild-type BM (Fig 4B). However, in wild-type mice receiving either wild-type or mutant BM, we did not find any differences in the numbers of blood T cells, neutrophils or monocytes (Fig 4E). While the number of IgD^-^IgM^+^ immature B cells was not reduced in the BM of SIRPα-mutants reconstituted with wild-type BM, IgD^+^IgM^hi^ F-II and IgD^+^IgM^lo^ F-I B cells were significantly reduced, as compared with that in wild-types receiving wild-type BM (Fig 4C), a phenotype remarkably similar to that seen in naïve SIRPα-mutant mice (Fig 2C). In contrast, these B cell subsets were similar in the BM of wild-type recipients of either wild-type or mutant BM (Fig 4F). Thus, SIRPα-signaling within the non-hematopoietic compartment appeared to be important to maintain circulating B cells and normal B cell maturation in the BM. Although very little is known about factors regulating B cell maturation in the BM, and RANKL is unlikely to affect B cell maturation *per se* [9], we decided to investigate if SIRPα-mutant BM stromal cells showed a phenotype different from that of their wild-type counterparts by measuring stromal cell RANKL gene expression. Interestingly, the gene expression of RANKL was significantly reduced by more than 50% in SIRPα-mutant BM stromal cells cultured for 12 days, as compared to wild-type stromal cells (Fig 5). In marked contrast to that seen in the BM, and despite a drop in blood B cell numbers, SIRPα-mutant mice reconstituted with wild-type BM did not have a significantly reduced number of F-II or F-I B cells in the spleen (Fig 6A). However, while blood B cell numbers and B cell maturation in the BM appeared normal in wild-type recipients of mutant BM, splenic F-II or F-I B cell numbers were reduced to a similar extent as that seen in naïve SIRPα-mutant mice (Fig 6B).

## Discussion

In the present study, we show that lack of SIRPα signaling in mice resulted in a reduced number of mature B cells in the BM, blood and spleen of adult mice, which was associated with an increased apoptosis among maturing B cells in BM or spleen. B cells do not express any significant levels of SIRPα, but by using BM chimeric mice we could show that non-hematopoietic SIRPα signaling was required to maintain normal numbers of mature B cells in BM and blood, whereas hematopoietic SIRPα signaling was required to maintain the same B cell subset in the spleen.

Homeostatic mechanisms regulate the numbers of peripheral B cells, which has been investigated for several decades. Throughout life, the BM continuously generate B cells in numbers that largely exceed what is required to maintain the pools of B cells in the periphery [31]. The latter is emphasized by the finding that about 30% of the normal number of BM precursors is enough to maintain a normal size of the peripheral B cell-pool [31]. The general dogma has been that immature B cells migrate via the blood to the spleen, where final maturation into follicular or marginal zone B cells takes place [1,2]. However, it has more recently been suggested that B cells mature into recirculating follicular B cells simultaneously in both the spleen and the BM [6]. It is therefore interesting to note, that SIRPα-mutant mice had a reduced number of IgD^+^IgM^hi^ semi-mature/F-II B cells and IgD^+^IgM^lo^ mature/F-I B cells in the BM, as well as a reduced number of the F-II and F-I B cell subsets in the spleen. In marked contrast to the deficiency among the F-II and F-I B cell populations in the BM of SIRPα-mutant mice, we found no decrease among the early Hardy fraction A-D or immature BM B cells. Together, this therefore suggests that lack of SIRPα signaling primarily results in an impaired B cell maturation in both the BM and the spleen. In the mouse, follicular B cells in the spleen as well as mature/recirculating B cells in the BM have a half-life of 4–5 months, depend on the BM for their renewal, and are supposed to be in equilibrium [32]. Thus, the reduction in blood B cells numbers in SIRPα-mutant mice probably reflects a combination of a reduced generation of immature B cells in the BM together with a strongly impaired maturation of B cells in both the BM and the spleen.

The mechanisms that regulate early B cell differentiation are fairly well described but less is known regarding the regulation of B cell maturation. We have previously shown that peripheral B cells do not express SIRPα [24], which is confirmed by the findings in the present study where Gr-1^-^B220^+^CD19^+^CD43^-^ BM B cells and splenic FoB cells were also shown to lack SIRPα expression. Thus, the B cell phenotype of SIRPα-mutant mice must depend on a requirement for SIRPα signaling in other cells of the BM or splenic environments. Interestingly, we found that the blood B cell numbers were normal in mutant → wild-type BM chimeras, but significantly reduced in wild-type → mutant chimeras. These findings therefore suggest that SIRPα signaling in the non-hematopoietic compartment is required to maintain normal B cell numbers in blood. A similar strong dependence of non-hematopoietic SIRPα signaling was observed to mediate maintenance of F-II and F-I B cells in the BM of chimeric mice. However, in contrast to that in naïve mutant mice, the numbers of immature BM B cells were not affected in these chimeric mice, which could suggest that the reduction of this B cell subset in naïve mice requires lack of SIRPα signaling in both the hematopoietic and non-hematopoietic compartments. Strikingly, and in marked contrast to that in the BM and blood, we found that the ability to maintain a normal number of splenic follicular B cells (F-II and F-I subsets) appeared to require SIRPα in hematopoietic cells.

It is well known that stromal cells play a pivotal role in maintaining homeostasis and functionality in both primary and secondary lymphoid organs [11]. Our recent findings strongly supports that SIRPα signaling is required for normal stromal cell function, since BM stromal cells express SIRPα, and SIRPα tyrosine phosphorylation is required for BM stromal cell osteoblastic differentiation and function [25]. In addition, we have previously reported that SIRPα-mutant mice have a marked reduction of the gp38^+^ FRC stromal cell population in their spleens, which contributes to reduced mRNA levels of CCL-21, CCL-19 and IL-7 in spleens of these mice [24]. Contributing to the overall signs of a stromal cell phenotype in SIRPα-mutant mice is our present finding that BM stromal cells of mutant mice have a strongly reduced expression of RANKL following *in vitro* culture, as compared with that in wild-type stromal cells. B cells express receptor activator of NF-κB (RANK) [33], which binds to RANKL [34]. However, B cell-specific deletion of RANK does not affect B cell differentiation, maturation or function [9], making the reduction in RANKL expression less likely to explain the impaired maturation of B cells in SIRPα-mutant mice. We previously found that the reduction of splenic gp38^+^ FRC stromal cells of SIRPα-mutant mice was due to lack of hematopoietic SIRPα signaling [24]. Interestingly, FRCs are also present in the B cell follicles, albeit at lower density than that in the T cell areas of the spleen [10]. In addition, recent findings have demonstrated that the presence of FRCs, but not that of FDCs, is required to maintain normal numbers of mature B cells in secondary lymphoid organs by providing BAFF [16]. Therefore, since hematopoietic SIRPα was required to maintain normal numbers of mature splenic B cells, it is possible that the reduced numbers of FRCs in SIRPα-mutant spleens could explain the reduced numbers of F-II and F-I B cells reported in the present study.

B cell survival is regulated by a balance between pro-survival and pro-apoptotic proteins in their cytoplasm [28]. One possibility could therefore be that there is a reduced survival of mature B cells in SIRPα-mutant mice. Although we were unable to show any difference in the pro-survival protein Bcl-2 or the pro-apoptotic protein BIM at the gene expression level when comparing sorted splenic FoB cells of wild-type and mutant mice, we found a significant increase in BIM at the protein level in maturing BM or splenic B cells in SIRPα-mutant mice. In addition, we found an increased apoptosis in these B cell subsets, which could explain the reduced numbers of mature B cells in SIRPα-mutant mice. IL-7 has been suggested to control the survival of follicular B cells via an indirect mechanism, since mature B cells do not express IL-7R [14]. Instead, Patton *et al*. suggested that a hematopoietic, IL-7Rα^+^, Rag1-dependent non-B cell is required for homeostasis of follicular B cells in the spleen [14]. Our previous findings are therefore interesting, which showed that the IL-7 gene expression is reduced in spleens of naïve SIRPα-mutant mice and that normal IL-7 expression requires SIRPα signaling in the hematopoietic compartment [24]. Thus, another possible mechanism could be that lack of SIRPα signaling in the hematopoietic compartment of the spleen results in an impaired IL-7-mediated support of follicular B cell homeostasis. This hypothesis is supported by our present findings of an increased apoptosis in splenic follicular B cells.

As already mentioned, although the factors regulating B cell maturation in the BM is not well understood, it has been suggested that the maintenance of mature/recirculating B cells in the BM depend on the survival factor macrophage migration–inhibitory factor (MIF) produced by BM dendritic cells [30]. We have previously shown that SIRPα-mutant mice have a reduced number of conventional CD4^+^CD11b^+^ dendritic cells in the spleen [23]. This phenotype requires SIRPα signaling in the hematopoietic compartment since it is maintained in mutant → wild-type BM chimeras, but not in wild-type → mutant chimeras [23]. Thus, given the fact that the reduced numbers of F-II and F-I B cells in the BM of SIRPα-mutant mice was dependent on SIRPα signaling within the non-hematopoietic, but not the hematopoietic compartment, it seems less likely that the BM phenotype of mutant mice could be due to a relative lack of dendritic cells.

In conclusion, we have shown that SIRPα signaling is required to maintain normal numbers of B cells in the BM, blood and spleen, and that lack of SIRPα signaling results in an increased apoptosis during B cell maturation. Surprisingly, the requirement for SIRPα signaling is different for these three tissues, such that non-hematopoietic SIRPα was found to be important to regulate the number of mature B cells in BM and blood, while hematopoietic SIRPα was required in the spleen. Thus, these findings show that slightly different mechanisms regulate mature B cell numbers in these tissues and that SIRPα is involved in two different ways in regulating B cell maturation and survival.

## Supporting Information

S1 Fig Bone marrow cells in SIRPα-mutant mice. (A) Total number of BM cells in femur and tibia of wild-type (Wt–open bars) or SIRPα-mutant mice (Mut–black bars) at increasing age. (B) Numbers of early B cells in BM of 12 weeks old wild-type (open bars) or SIRPα-mutant mice (black bars). Early BM B cells were identified as Hardy fractions A (B220^+^ IgM^-^ CD43^hi^ CD19^-^), B-C (B220^+^ IgM^-^ CD43^hi^ CD19^+^), or D (B220^+^ IgM^-^ CD43^lo^ CD19^+^). Data are means ± SEM for 3–6 mice/group.
(TIF)

S2 Fig Lack of SIRPα expression in bone marrow B cells or splenic FoB cells. (A) Expression of SIRPα was analyzed using flow cytometry in BM cells by gating on B cells (B220^+^ IgM^+^ CD43^-^ cells or B220^+^CD19^+^ CD43^-^ Gr-1^-^ cells) or neutrophils (Gr-1^+^ CD43^+^ cells). (B) Expression of SIRPα was analyzed using flow cytometry in splenic FoB cells (B220^+^ CD23^hi^ CD21^lo^ cells). Grey histograms represents cells incubated with the Alexa 488-conjugated anti-SIRPα-mAb P84, black lines represent cells incubated with Alexa 488-conjugated rat IgG1 isotype control mAb.
(TIF)

S3 Fig Normal cell cycle activity in BM or splenic B cells of SIRPα-mutant mice. The fractions of B cells in G0/G1 or G2-M phase were determined in the indicated BM B cell subsets (A-D) or splenic B cell subsets (E-F), using the Vybrant DyeCycle Ruby Stain by gating on specific B cell subsets as described in the legends to Figs 2 and 3. Data are means±SEM for 5 mice/group.
(TIF)
