# Supplementary figures and images for: Non-Hematopoietic and Hematopoietic SIRPα Signaling Differently Regulates Murine B Cell Maturation in Bone Marrow and Spleen
Source: PLoS One. 2015 Jul 29;10(7):e0134113. doi: 10.1371/journal.pone.0134113 (PMC4519279; doi:10.1371/journal.pone.0134113)

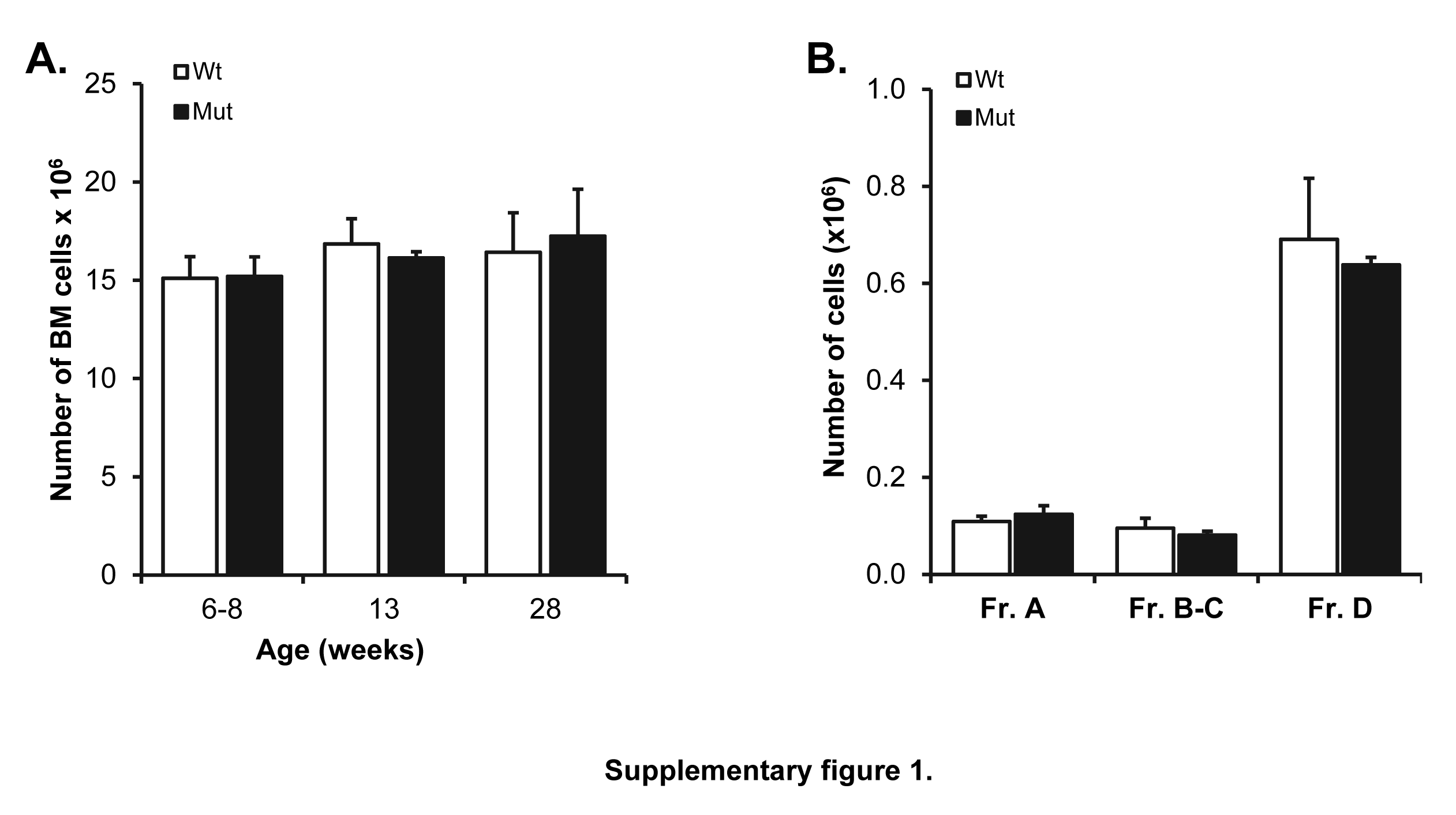

Supplement: S1 Fig — (A) Total number of BM cells in femur and tibia of wild-type (Wt–open bars) or SIRPα-mutant mice (Mut–black bars) at increasing age. (B) Numbers of early B cells in BM of 12 weeks old wild-type (open bars) or SIRPα-mutant mice (black bars). Early BM B cells were identified as Hardy fractions A (B220+ IgM- CD43hi CD19-), B-C (B220+ IgM- CD43hi CD19+), or D (B220+ IgM- CD43lo CD19+). Data are means ± SEM for 3–6 mice/group. (TIF) [file pone.0134113.s001.tif]

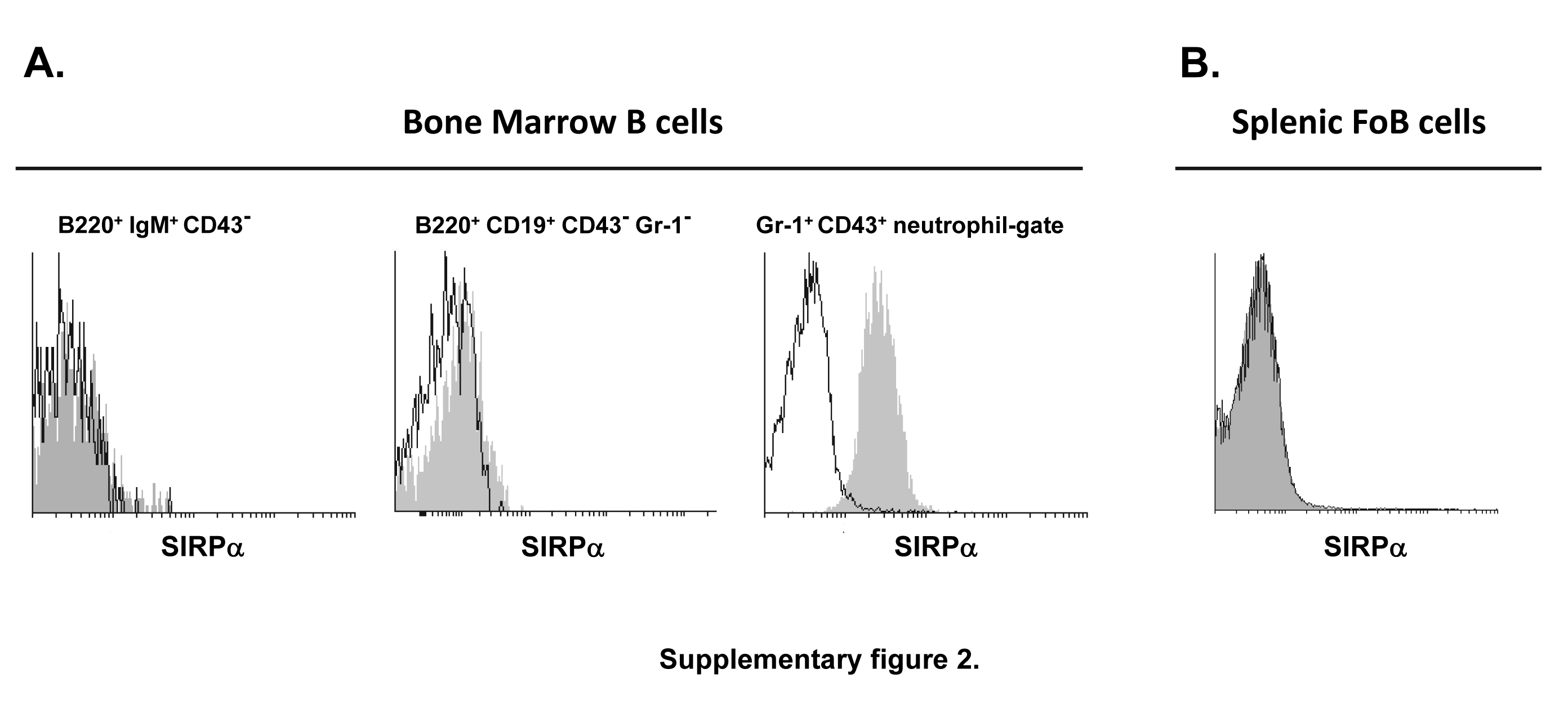

Supplement: S2 Fig — (A) Expression of SIRPα was analyzed using flow cytometry in BM cells by gating on B cells (B220+ IgM+ CD43- cells or B220+CD19+ CD43- Gr-1- cells) or neutrophils (Gr-1+ CD43+ cells). (B) Expression of SIRPα was analyzed using flow cytometry in splenic FoB cells (B220+ CD23hi CD21lo cells). Grey histograms represents cells incubated with the Alexa 488-conjugated anti-SIRPα-mAb P84, black lines represent cells incubated with Alexa 488-conjugated rat IgG1 isotype control mAb. (TIF) [file pone.0134113.s002.tif]

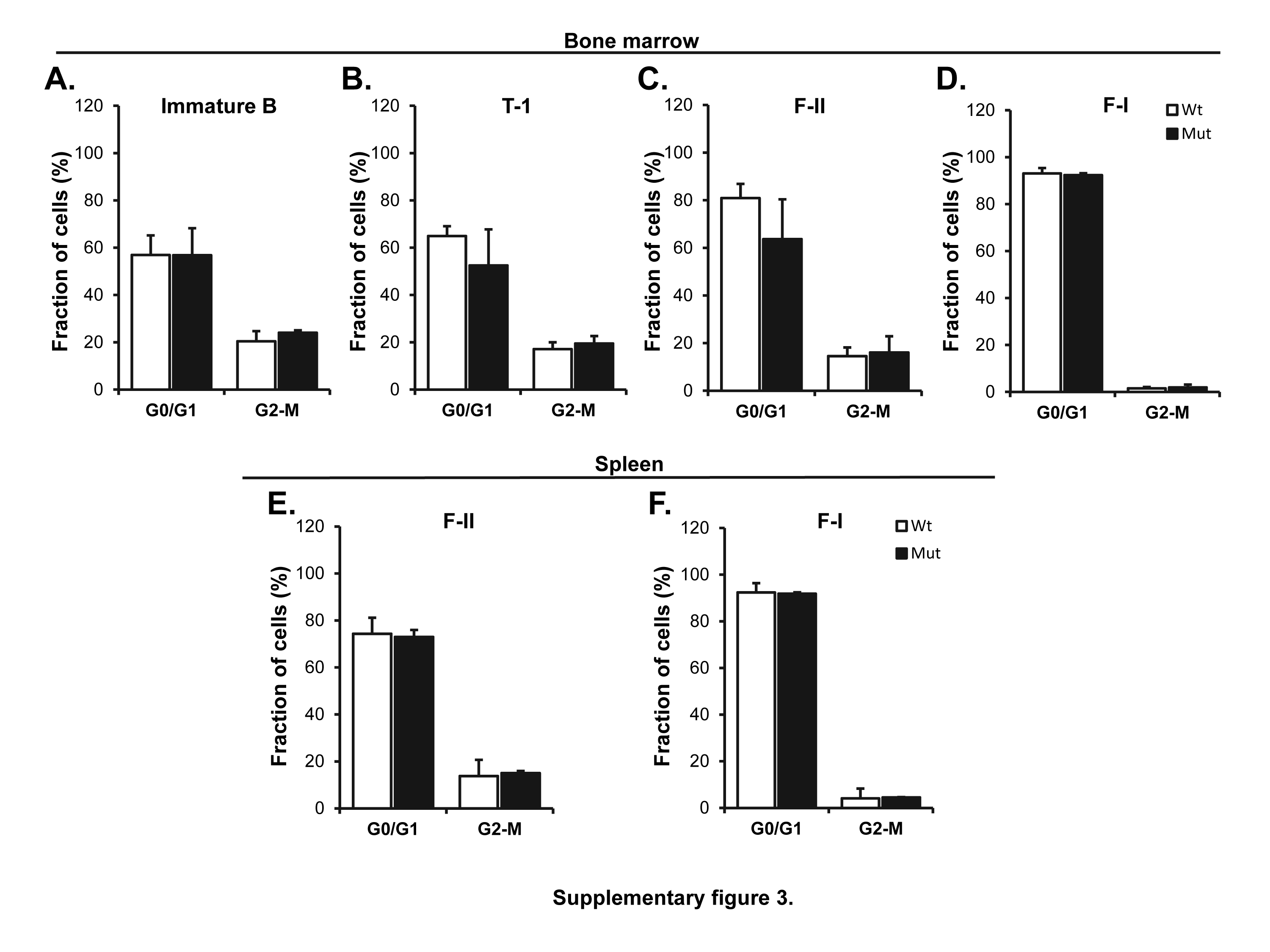

Supplement: S3 Fig — The fractions of B cells in G0/G1 or G2-M phase were determined in the indicated BM B cell subsets (A-D) or splenic B cell subsets (E-F), using the Vybrant DyeCycle Ruby Stain by gating on specific B cell subsets as described in the legends to Figs 2 and 3. Data are means±SEM for 5 mice/group. (TIF) [file pone.0134113.s003.tif]
